# Differentiating cervical metastatic lymphadenopathy and lymphoma by shear wave elastography

**DOI:** 10.1038/s41598-019-48705-0

**Published:** 2019-08-27

**Authors:** Soo Young Chae, Hye Na Jung, Inseon Ryoo, Sangil Suh

**Affiliations:** Department of Radiology, Korea University Guro Hospital, Seoul, 08308 Korea

**Keywords:** Diagnostic markers, Cancer imaging

## Abstract

Shear wave elastography (SWE) is a recent technological advance of ultrasonography (US) to assess tissue stiffness. The purpose of this study is to assess tissue stiffness of malignant cervical lymph nodes (LN) with SWE, to reveal diagnostic performance of SWE in differentiating metastatic LN from lymphoma, and to assess inter-observer agreement of SWE. We assessed 62 malignant LN (24 lymphomas and 38 metastatic LN) and their median speed was 6.34 m/s and median elasticity was 69.7 kPa. Add of SWE with conventional US improved diagnostic accuracy of differentiating metastasis from lymphoma (16.13, 8.07 and 11.3% for three radiologists). Kendall’s Coefficient of Concordance of three readers for analyzing SWE patterns was 0.86. SWE can be a useful tool to discriminate metastatic cervical LN from lymphoma with improvement of diagnostic accuracy when using with conventional US.

## Introduction

Metastasis and lymphoma are two common malignant diseases with head and neck involvement presented as enlarged cervical lymph nodes (LN). As an initial diagnostic imaging of choice for cervical lymphadenopathy (LAP), the role of conventional ultrasonography (US) is well established, and malignant cervical LN with typical image findings are not too difficult to differentiate from benign LN. However, health professionals often encounter challenging cases with overlapping imaging features in differentiating metastasis from lymphoma only with conventional B-mode US and even with Doppler US^1–3^. On gray scale US, metastatic LN are usually hypoechoic and round with loss of hilum. Internal necrosis of metastatic nodes can be seen as either echogenic focus or cystic or low echoic area depending on the type of necrosis. Lymphomatous LN also tend to be round and hypoechoic with loss of hilum as metastatic LN do, but they tend to show intranodal reticulation and have sharp margins. On Doppler US, either metastatic LN or lymphoma usually show peripheral or mixed patterns of internal vascularity^4–6^. In daily practice, radiologists may be confused to differentiate metastasis from lymphoma when they examine a round heterogeneous echoic cervical LN with loss of hilum, without definite necrotic area and showing internal mixed vascularity. In the special circumstances, peripheral T cell lymphoma usually have ill-defined margins because they first involve the marginal sinuses of the nodal cortex and then the medulla resulting blockage of the lymph flow and internal nodal necrosis^7^. Moreover, it is important to discriminate these two entities, as their prognosis and treatment are totally different. Therefore, advanced imaging techniques are needed to differentiate malignant LAP for better evaluation and treatment.

Shear wave elastography (SWE) is a recent technological advance of US which is able to assess tissue stiffness^8^. Qualitative and quantitative estimates of tissue elasticity from SWE have been used the evaluation of many different types of organs, including the breast, liver, prostate, thyroid glands, blood vessels, salivary glands, musculoskeletal structures, and cervical LN^9^. There are many studies that usefulness of US elastography as a new imaging biomarker in detecting malignant LN (including both metastatic LAP and lymphoma) from benign LAP^5,9–12^. However, there is no study assessing SWE for differentiating metastatic LN from lymphoma in the head and neck region, and there are only a few studies of researching lymphoma in cervical region on strain elastography^6,13^.

We hypothesized the pathologic differences between metastasis and lymphoma may differ tissue elasticity and influence qualitative and quantitative analysis of SWE. The purpose of this study is to assess tissue stiffness of malignant cervical LAP with SWE, to reveal diagnostic performances of the SWE in differentiating metastatic LAP from lymphoma and to assess inter-observer agreement of the technique.

## Results

### Analysis of SWE features

**Figure 1:**
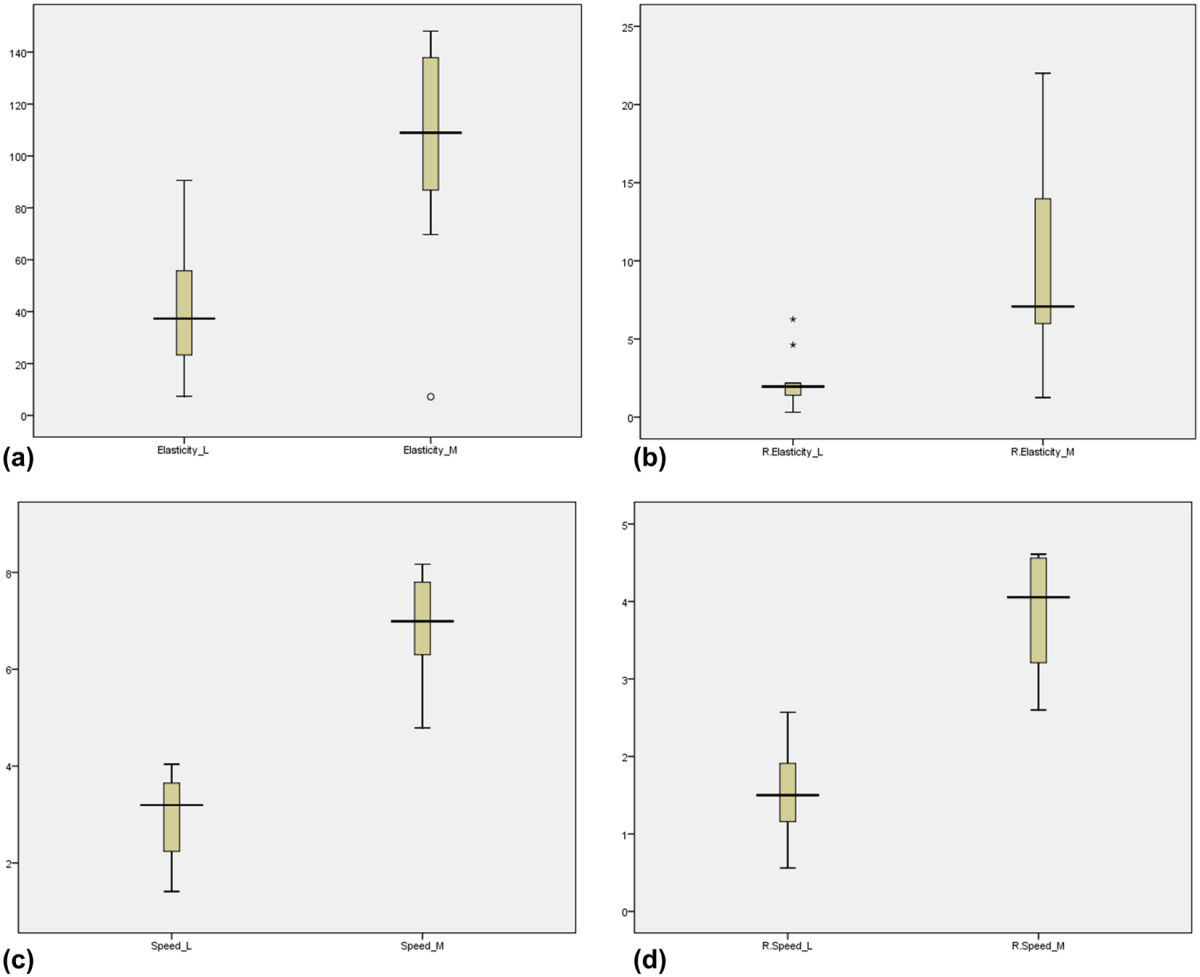
(**a~d**) Results of absolute and relative values of shear wave elastography. (**a**) Absolute elasticity of metastasis (Elasticity_M) was significantly higher than that of lymphoma (Elasticity_L). One case of metastasis from lung adenocarcinoma was an outlier. (**b**) Relative elasticity of metastasis (R. Elasticity_M) was significantly higher than that of lymphoma (R. Elasticity_L). Two cases of diffuse large B cell lymphoma were outliers. (**c**) Absolute speed of metastasis (Speed_M) was significantly higher than that of lymphoma (Speed_L). (**d**) Relative speed of metastasis (R. speed_M) was significantly higher than that of lymphoma (R. speed_L). Among these four plots, absolute speed has the least overlapping feature. Boxes and whiskers plots express medians, interquartile and overall ranges. The outlying values are plotted individually.

**Figure 2:**
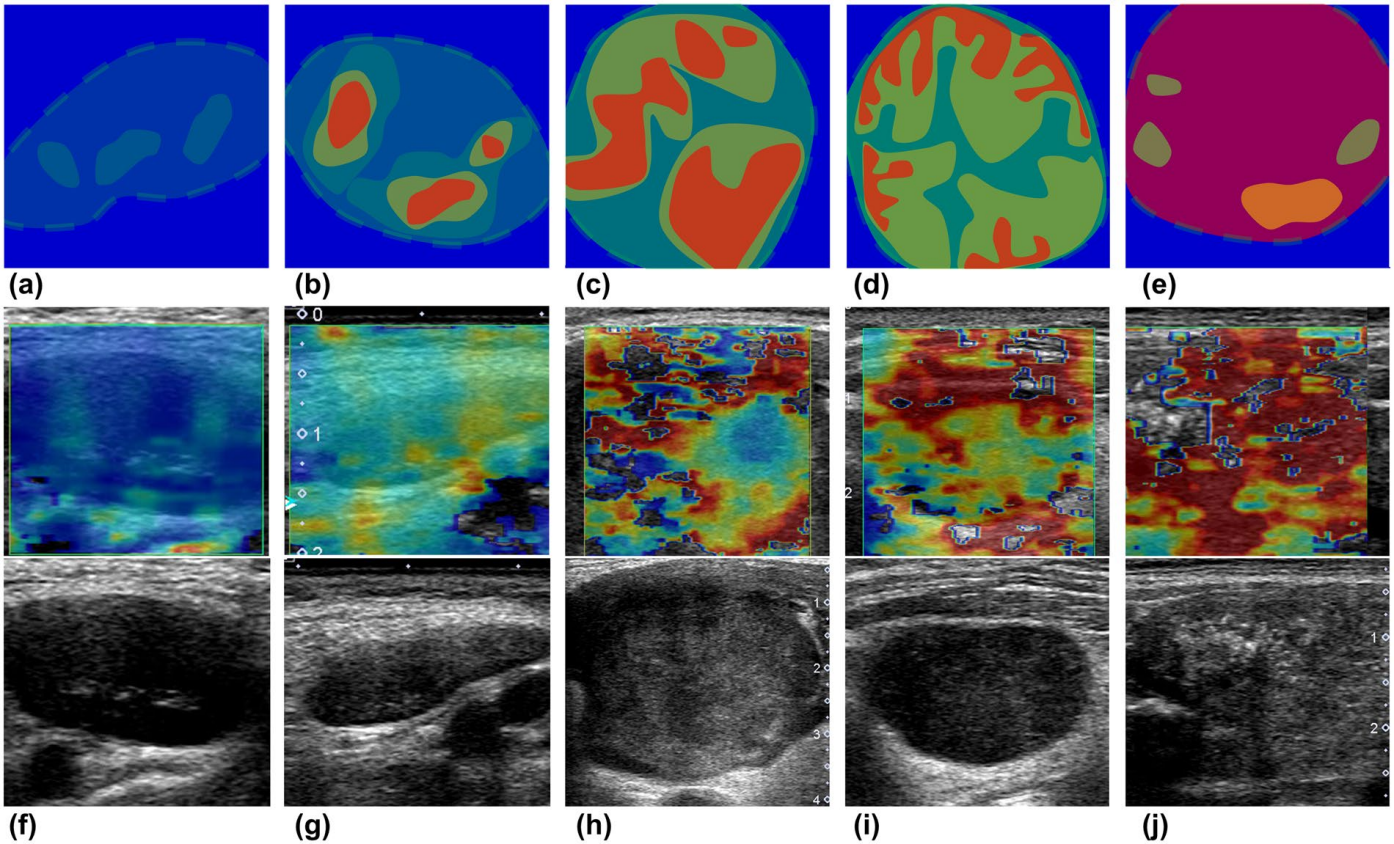
(**a~e**) Schematic drawings of five patterns shear wave elastography. We categorized total five SWE patterns of shear wave speed maps. In cervical lymph nodes (green dotted lines). (**a**) The pattern 1 nodes have absent or very small red (stiff) area; (**b**) the pattern 2 nodes have small scattered red areas, which mean total red area less than 45%; (**c**) the pattern 3 nodes have large red area, equal or more than 45%; (**d**) the pattern 4 nodes show peripheral red are and central green (soft) area, suggesting central necrosis; and (**e**) the pattern 5 nodes appear as almost red area with or without a green rim. (**f**~**j**) The examples of pattern analysis. (**f**) The pattern 1: diffuse large B cell lymphoma; (**g**) the pattern 2: T-cell/histiocyte rich large B cell lymphoma; (**h**) the pattern 3: metastatic squamous cell carcinoma from nasopharynx; (**i**) the pattern 4: metastatic adenocarcinoma from lung; and **(j**) the pattern 5: metastasis from papillary thyroid carcinoma

There were statistically significant differences between these two groups in the analysis of SWE pattern and both absolute value and ratio of elasticity and speed (Table 1 and Fig. 1). On SWE pattern analysis (Fig. 2), none of lymphoma had large red area (≥45%) suggesting stiffness (pattern 5) nor internal necrosis (pattern 4). More than half of metastatic LN (26/38, 68.42%) had large red area (pattern 3 or 5, 26/38, 68.42%) and internal necrosis (5/26, 19.2%). Absolute values and ratio of both elasticity and speed were significantly higher in metastatic LN than lymphoma (p < 0.001). Mean elasticity and speed of metastatic LAP were 94.87 kPa and 5.63 m/s, and those of lymphoma were 42.75 kPa and 3.49 m/s. Mean ratio of elasticity and speed of metastases were 9.08 and 2.84, and those of lymphoma were 2.57 and 1.56, respectively.

**Table 1:** SWE features of the LN.

SWE features	Metastasis (n = 38)	Lymphoma (n = 24)	P value
**SWE pattern**			
Absent or very small red area	6 (15.79%)	14 (58.33%)	<0.001
Small scattered red areas, red area <45%	4 (10.53%)	8 (33.33%)	
Large red area ≥45%	12 (31.58%)	2 (8.33%)	
Peripheral red are and central green area	2 (5.26%)	0 (0%)	
Red area with or without a green rim	14 (36.84%)	0 (0%)	
**SWE value (mean)**			
Elasticity (kPa) (range)	94.87 ± 41.70 (7.2–148.1)	42.75 ± 30.62 (7.2–127)	<0.001
Speed (m/sec) (range)	5.63 ± 1.99 (1.51–8.36)	3.49 ± 1.49 (1.41–7.01)	<0.001
Relative elasticity (range)	9.08 ± 7.62 (1.18–32.23)	2.57 ± 1.74 (0.31–6.65)	<0.001
Relative speed (range)	2.84 ± 1.40 (1.09–6.58)	1.56 ± 0.55 (0.56–2.57)	<0.001

### Diagnostic performance of SWE features

**Figure 3:**
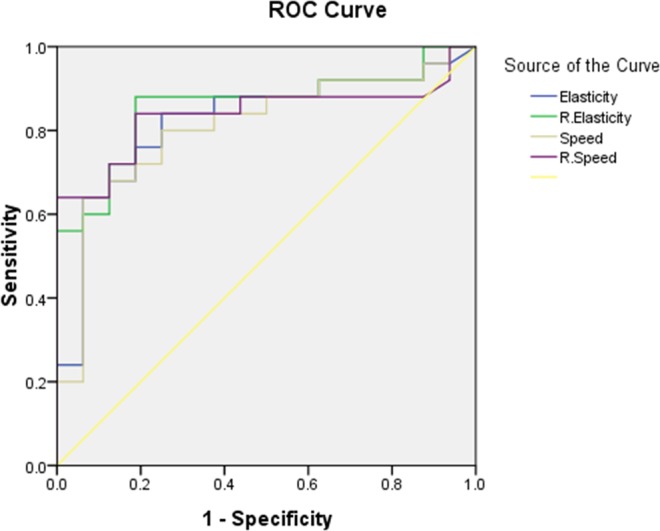
ROC curves of shear wave elastography. ROC analysis was performed to obtain the best value for differentiating metastasis from lymphoma. The AUC values by max elasticity (**a** blue line), relative elasticity (**a** green line), max speed (**a** gold line) and relative speed (**a** purple line) were 82.1, 80.8, 85.8 and 83.33, respectively. The cut-off vaule of relative speed had the largest AUC and it was 1.915 (p < 0.001).

Diagnostic performances of SWE for differentiating two groups with elasticity, relative elasticity, speed and relative speed were all good (Table 2). Among them, relative speed had the largest area under the curve (Fig. 3) with 1.915 of optimal cut-off and its accuracy, sensitivity, specificity, positive predictive value and negative predictive value were 83.33%, 84%, 81.25%, 88% and 76.47%, respectively. (p < 0.001).

**Table 2:** ROC analysis in differentiating metastasis from lymphoma.

Parameter	Cut-off value	Sensitivity (%)	Specificity (%)	PPV (%)	NPV (%)	Accuracy (%)	AUC (95% CI)	p value
Elasticity (kPa)	≥52.8	84	75	83.33	73.68	79.59	82.1 (68.6–95.6)	0.001
Speed (m/sec)	≥4.19	80	75	86.21	70.59	80.43	80.8 (66.9–94.6)	<0.001
Relative elasticity	≥3.455	88	81.25	85.71	78.95	82.98	85.8 (74.0–97.5)	0.001
Relative speed	≥1.915	84	81.25	88	76.47	83.33	83.9 (71.1–96.6)	<0.001

### Added value of diagnostic performance of SWE with conventional US

**Figure 4:**
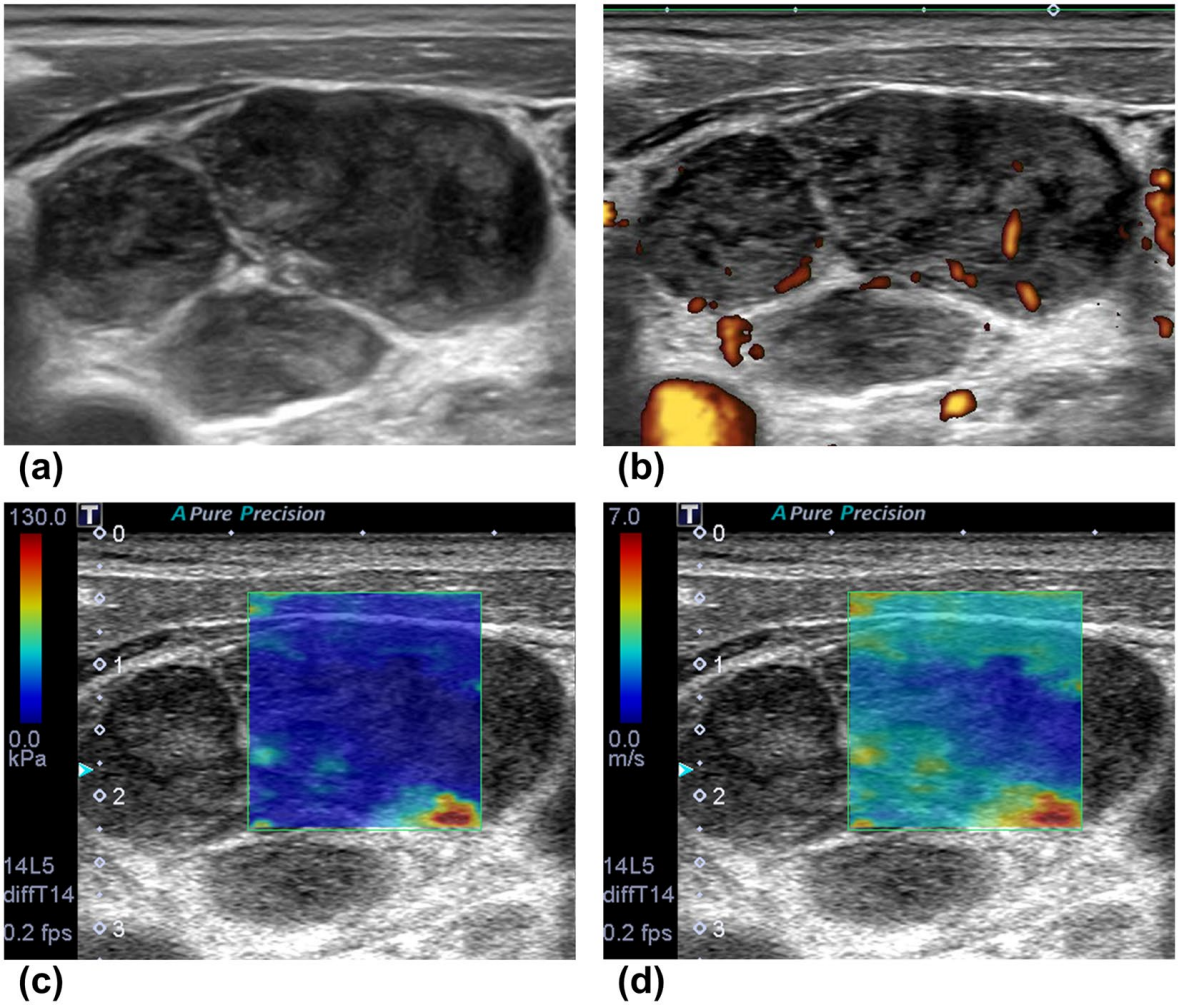
The case of diffuse large B cell lymphoma. On conventional ultrasound (**a**: 2D echo, **b**: power Doppler US), multiple round lymph nodes have heterogeneous echogenicity and show indistinct border with internal mixed vascularity. Two readers diagnosed these lymph nodes as metastasis with 2D and Doppler US. After evaluation with shear wave elastography (**c**: the elasticity map, d: the speed map), all readers classified the nodes as pattern 2, and the two readers corrected their diagnosis as lymphoma. Maximum elasticity, relative elasticity, maximum speed and relative speed were 30.4 kPa, 1.32, 3.16 m/s and 1.16, respectively.

**Figure 5:**
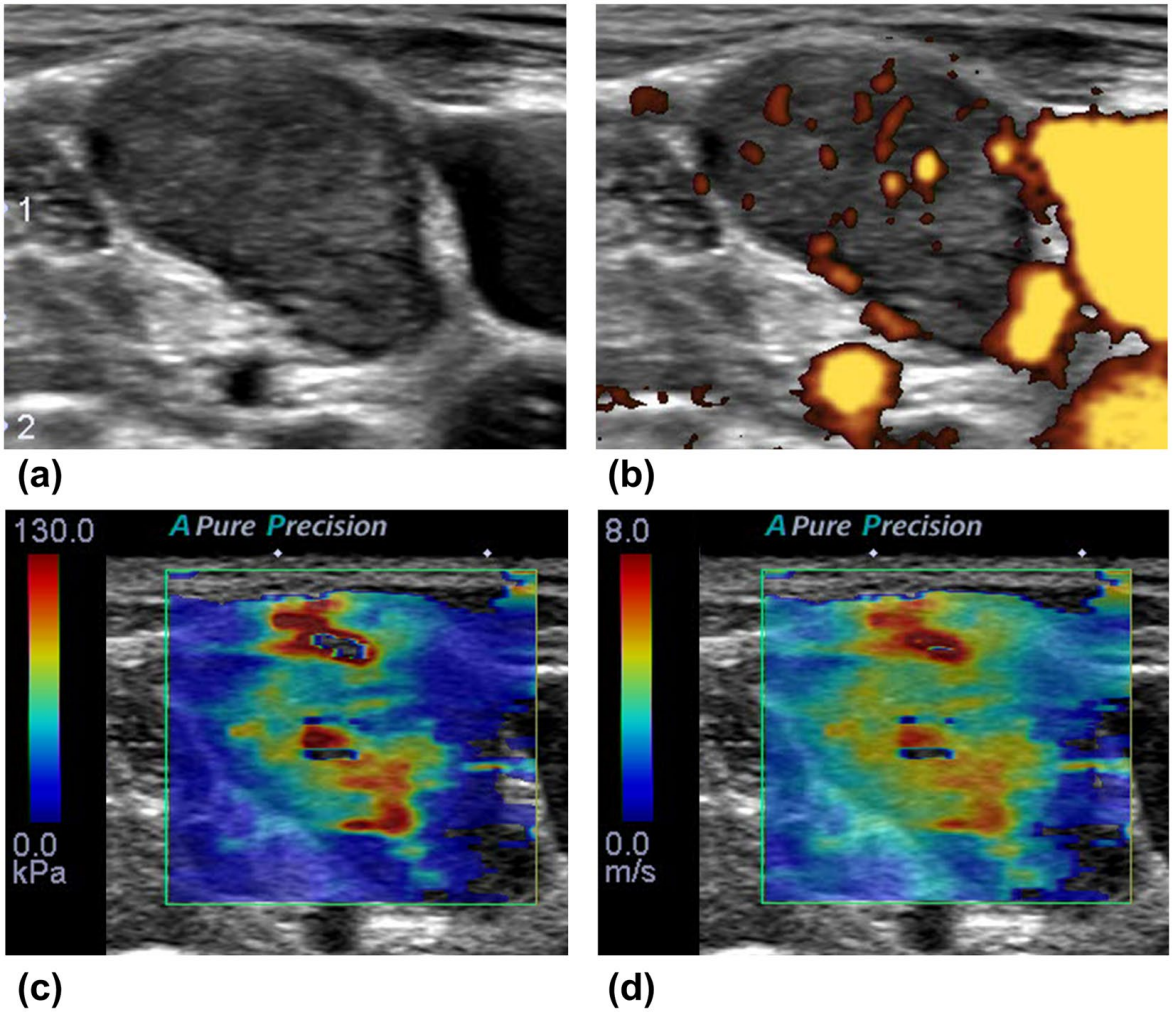
The case of metastatic squamous cell from lung. On conventional ultrasound (**a**: 2D echo, **b**: power Doppler US), an oval lymph node with loss of hilum has relatively homogeneous high internal echogenicity and shows regular border with internal mixed vascularity. Two readers diagnosed these lymph nodes as lymphoma with 2D and Doppler US. After evaluation with shear wave elastography (**c**: the elasticity map, **d**: the speed map), all readers classified the nodes as pattern 3, and the two readers corrected their diagnosis as metastasis. Maximum elasticity, relative elasticity, maximum speed and relative speed were 92.3 kPa, 7.1, 7 m/s and 3.44, respectively.

Diagnostic performance for differentiating metastatic LN from lymphoma by three readers are presented in Table 3. SWE showed tendencies to improve diagnostic accuracy of differentiating metastasis from lymphoma. For all three radiologists, diagnostic accuracies of SWE with conventional US (83.87%, 80.65% and 80.65% for reader 1, 2 and 3) were higher than that of only conventional US (67.74%, 72.58% and 69.35% for reader 1, 2 and 3). With SWE pattern analysis on conventional US, three readers corrected their initial diagnosis metastasis to lymphoma in 13 of total 72 diagnosis of lymphoma (24 lymphomas for each three readers) (Fig. 4). Also, in 24 of total 114 diagnosis of metastasis (38 metastatic LN for each three readers), they changed their decision correctly (Fig. 5).

**Table 3:** Added value of diagnostic performance of SWE with conventional US between observers in differentiating metastasis from lymphoma.

Diagnostic performance	2D + Doppler US	2D + Doppler US + SWE
**Reader 1**		
Accuracy (%)	67.74 (42/62)	83.87 (52/62)
Sensitivity (%)	71.05 (27/38)	81.58 (31/38)
Specificity (%)	62.50 (15/24)	87.50 (21/24)
Positive predictive value (%)	75.00 (26/33)	91.18 (31/34)
Negative predictive value (%)	57.69 (15/26)	75.00 (21/28)
**Reader 2**		
Accuracy (%)	72.58 (45/62)	80.65 (50/62)
Sensitivity (%)	65.79 (25/38)	71.05 (27/38)
Specificity (%)	83.33 (20/24)	95.83 (23/24)
Positive predictive value (%)	86.21 (25/29)	96.43 (27/28)
Negative predictive value (%)	60.61 (20/33)	67.65 (23/34)
**Reader 3**		
Accuracy (%)	69.35 (43/62)	80.65 (50/62)
Sensitivity (%)	63.16 (24/38)	76.32 (29/38)
Specificity (%)	79.17 (19/24)	87.50 (21/24)
Positive predictive value (%)	82.76 (24/29)	90.63 (29/32)
Negative predictive value (%)	57.58 (19/33)	70.00 (21/30)

### Inter-observer agreement

Agreement on analysis of SWE patterns between three readers was evaluated with Kendall’s Coefficient of Concordance. Kendall’s Coefficient of Concordance can range from 0 to 1. The higher the value of Kendall’s, the stronger the association and the value of three readers was 0.86.

### Analysis of gray scale and Doppler US features

In the analysis of gray scale US, echogenicity, presence of hilum and nodal border could differentiate metastasis from lymphoma significantly (p = 0.019, <0.01 and 0.008 for echogenicity, presence of hilum and nodal border respectively) (Table 4). There are 21 out of 38 metastatic LN (55.26%) showing inhomogeneous echogenicity, in contrast to only 6 out of 24 lymphoma (25%) showing inhomogeneous echogenicity. In terms of presence of hilum, the majority of metastatic LAP presented absence of hilum (35/38, 92.11%), whereas 10 out of 24 lymphoma (41.67%) had hilar structure. Most of lymphoma showed regular nodal border (20/24, 83.33%), but a half of metastatic LN (19/30. 50%) had irregular border.

**Table 4:** Gray scale and Doppler US features of the LN.

US features		Metastasis (n = 38)	Lymphoma (n = 24)	P value
Long diameter (mean ± SD)		2.40 ± 0.86	2.59 ± 0.93	0.429
Short diameter (mean ± SD)		1.46 ± 0.57	1.59 ± 0.72	0.435
Shape	Oval	7 (18.42%)	6 (25%)	0.535
	Round	31 (81.58%)	18 (75%)	
Necrosis	Present	4 (10.53%)	2 (8.33%)	0.776
	Absent	34 (89.47%)	22 (91.67%)	
Echogenicity	Homogeneous	17 (44.74%)	18 (75%)	0.019
	Inhomogeneous	21 (55.26%)	6 (25%)	
Calcification	Present	3 (7.89%)	0 (0%)	0.158
	Absent	35 (92.11%)	24 (100%)	
Hilum	Present	3 (7.89%)	14 (58.33%)	<0.001
	Absent	35 (92.11%)	10 (41.67%)	
Nodal border	Regular	19 (50%)	20 (83.33%)	0.008
	Irregular	19 (50%)	4 (16.67%)	
Vascular distributions	Avascular	5 (13.16%)	0 (0%)	<0.001
	Peripheral	16 (42.11%)	2 (8.33%)	
	Central	2 (5.26%)	10 (41.67%)	
	Mixed	15 (39.47%)	12 (50%)	
Number of vascular pedicles	Absent	5 (13.16%)	0 (0%)	0.043
	A single	1 (2.63%)	1 (4.17%)	
	A few (2~4)	16 (42.11%)	5 (20.83%)	
	Multiple (>5)	16 (42.11%)	18 (75%)	

On Doppler US, vascular distribution and number of vascular pedicles differed significantly between the two groups (p value: < 0.001 and 0.043) (Table 4). Majority of LN had peripheral or mixed vascular pattern on both metastatic LAP (31/38, 81.58%) and lymphoma (14/24, 58.33%). However, lymphoma had more mixed vascular distribution (12/24, 50%) than that of metastasis (15/38, 39.47%). Vice versa, metastasis had more peripheral vascular distribution (16/38, 42.11%) than that of lymphoma (2/24, 8.33%). In terms of number of vascular pedicles, lymphoma tends to have multiple vascular pedicles (18/24, 75%) than metastasis does (16/38, 42.11%).

## Discussion

This is the first study to investigate the added value of the diagnostic accuracy with SWE for differentiating malignant cervical LAP. The results of our study show that SWE, a recent sonographic technique, allows substantial differentiation of metastatic LN from lymphoma and prove that SWE can be a potential discriminator of these two diseases.

In the last decade, various clinical applications for SWE been under investigation in head and neck. SWE displays real time tissue stiffness and provides quantitative assessment with elasticity (kPa) and speed (m/sec). A part of the tissue is deformed by a ‘push pulse’, the velocity of the shear waves propagating within the tissue is detected, and the stiffness of the tissue is assessed based on the detected shear velocity to both m/s and kPa. If an object such as a tumor is present in the tissue, the shear velocity in that area differs from the shear velocity in the surrounding areas. If the object is stiffer than the surrounding tissues, the shear waves propagate faster, and if softer, the shear waves propagate slower. The propagating shear waves are detected by transmitting tracking pulses.

SWE has been suggested as an acceptable imaging technique for diagnosing malignant cervical nodes^14–21^. In a meta-analysis of eight studies, malignant nodes are stiffer (higher elasticity and higher speed) than benign nodes and the final results of the studies conclude that SWE for diagnosing malignant cervical LN had a summary sensitivity of 81% and a specificity of 85%, and the area under the curve was 0.88^22^. In subsequent studies of SWE, regarding a relatively large number of patients^12^, metastasis from nasopharyngeal carcinoma^23^ and lymphoma in pediatric patients^24^, consistently show more stiffness of malignant LN than benign LN. Although most of these studies implement acoustic radiation force impulse imaging (ARFI) and Supersonic shear imaging (SSI), there is a one study used Toshiba SWE (T-SWE) for evaluating cervical LAP, the same technique that we used in this study^24^. Cut-off values for differentiating malignant LN varied from 1.9 to 3.34 m/s by Virtual Touch Tissue Imaging and Quantification (VTIQ) using ARFI and 31 to 45 kPa using SSI. In our study, all LN are malignant and median speed was 6.34 m/s and median elasticity was 69.7 kPa, which is consistent with the previous studies. Up to date, no studies have been suggested comparison of SWE parameters with these three SWE techniques, especially in nodal disease, without agreement of reference standards for malignant LN. He *et al*.^25^ suggested that VTIQ using ARFI and T-SWE are comparable and reproducibility of VTIQ and T-SWE are both favorable in diagnosing thyroid nodules.

The reason why malignant LN have tendency to show higher tissue stiffness is associated altered tissue composition and structure. For metastatic LN, cortex were damaged and thickened for proliferation and cornification of cancer cells and interstitial cells^26^ and these alteration may produce a great deal of keratin or fibrin in the early stage before morphological changes. Especially, metastatic LN originating from thyroid papillary carcinoma have microcalcifications in result in high stiffness^27^. In contrast to metastatic LN, desmoplastic reaction is limited and rarely observed in non-Hodgkin lymphoma, nodular lymphocyte predominant Hodgkin’s lymphoma and lymphocyte depleted Hodgkin’s lymphoma. In these diseases, consistency of LN is determined by dense ‘folding’ of rapidly proliferating cells. We considered these pathologic differences may differ the result of tissue elasticity of metastatic and lymphomatous LN, resultant of different SWE value of two diseases. The advanced subtypes of nodular sclerosis and mixed-cell of Hodgkin’s lymphoma are exceptional, because they usually have desmoplastic reaction, and this feature may be the cause of difficulty to differentiate them from metastatic LN^28^. However, in our study, two mixed cell type Hodgkin’s lymphoma had SWE pattern 1 (absent or very small red area) so all three radiologists could diagnose them correctly.

SWE provides absolute quantification of tissue stiffness without effort to free-hand compression, which means less operator dependent and more reproducible. In our study, SWE improved diagnostic accuracy of differentiating metastasis from lymphoma. Add of SWE with conventional US increase 16.13, 8.07 and 11.3% of accuracy for three radiologists. Reader 1, the least experienced neuroradiologist, gained the greater accuracy owing to SWE. Furthermore, it is more time saving using SWE pattern analysis without drawing ROI on the lesion in the clinical practice for fast spot diagnosis and its correlation between the readers was good in our results.

We have several some limitations. First, this was a retrospective study and subject to the inherent limitations of such a study design. Second, our sample size was relatively small, with evaluations made at a single institution. However, our result had significance in differentiating metastatic LN from lymphoma and we believe that these may provide clinical importance and it could be an important background data for future large-scale prospective studies. Third, we included various malignant LN with different primary malignancies and different subtypes of lymphoma, and this could not reflect individual pathologic characteristics, which can lead to different tissue elasticity. Despite including various pathology, there is a benefit of SWE in differentiating cervical metastasis and lymphoma. Further studies will be needed to define the added value of SWE in the diagnosis of each individual pathology. Lastly, we measured the highest portion of the LN, but this may not represent whole pathology of the LN. However, we also evaluated SWE patterns and this method may overcome the limitation of quantitative assessment.

In conclusion, our study showed that SWE can be a useful tool to discriminate metastatic cervical LN from lymphoma with improvement of diagnostic accuracy when using with conventional US. Furthermore, SWE may improve accuracy without too much time consuming for especially less-experienced radiologists.

## Materials and Methods

### Patient selection and study population

This study was approved by the institutional review board of Korea University Guro Hospital [2018GR0278] and the requirement for informed consent was waived. A total of 434 consecutive patients with cervical LAP underwent US guided core needle biopsy for pathologic evaluation between December 2016 and August 2018. Imaging and clinical data were retrospectively obtained from the picture archiving and communication system and the computerized medical records at our hospital. We included patients with pathologically proven metastasis and lymphoma in cervical LNs who underwent SWE technique in the US evaluation.

A total of 62 nodes in 62 patients with pathologically proven metastatic LAP (n = 38) or lymphoma (n = 24) were included in this study. Among the patients with metastatic LAP, 32 were male and 6 were female, and their mean age was 62.3 ± 14.9 years (range: 18–83). Pathologic subtypes were squamous cell carcinoma (n = 18, from hypopharynx [n = 3], nasopharynx [n = 3], esophagus [n = 2], lung [n = 2], larynx [n = 2], oropharynx [n = 2], skin [n = 1], uterine cervix [n = 1] and unknown origin [n = 2]); adenocarcinoma (n = 10, from lung [n = 7], colon [n = 1], stomach [n = 1] and gall bladder [n = 1]); small cell carcinoma (n = 4, from lung [n = 1] and nasal cavity [n = 1]); neuroendocrine carcinoma (n = 1, from rectum); serous carcinoma (n = 1, from ovary); mucinous carcinoma (n = 1, from ovary); dysgerminoma (n = 1, from ovary); papillary carcinoma (n = 1, from thyroid); and unknown metastatic carcinoma (n = 1). Among the 24 patients with lymphoma, 11 were male and 13 were female, and their mean age was 63.7 ± 14.1 years (range: 26–84). Pathologic diagnosis were subdivided into non-Hodgkin’s lymphoma (n = 22, diffuse large B cell lymphoma [n = 14]; peripheral T cell lymphoma [n = 4]; angioimmunoblastic T cell lymphoma [n = 2]; mature T cell lymphoma [n = 1]; and T-cell/histocyte rich large B cell lymphoma [n = 1]) and Hodgkin’s lymphoma (n = 2).

### Ultrasonography and core needle biopsy

All 62 patients underwent gray scale US, Doppler US and SWE with high-resolution ultrasonography units and high-frequency linear transducers (Aplio 500, 4.2 ~ 14.0 MHz, Canon Medical Systems Corp., Japan). All studies were performed in a supine position and the patient’s head was turned away from the site of examination with hyperextension. Two head and neck radiologists (S.S. and I.R. 19 and 7 year of experience in this field) performed US exams and US guided core needle biopsy. Doppler US were performed with standardized Doppler parameters set to high sensitivity and a low wall filter to allow detection of blood vessels with weaker blood flow (frame rate, 7–9/sec; scale, 4.9–6.1 cm/sec; pulse repetition frequency, 13.7–15.6 kHz). The real-time shear wave map were demonstrated on multiple LN including biopsied one. The elasticity range was set to 0~130 kPa, and the speed range was set to 0~7 or 8 m/sec.

US-guided core needle biopsy on the largest pathologic lymph node using a disposable 18-gauge gun biopsy needle (TSK Ace-cut, Create Medic, Yokohama, Japan; or Angiotech, Medical Device Technologies, Gainesville, FL, USA).

### Image analysis and interpretation

All images were analyzed by four head and neck radiologists (S.S., I.R., H.J. and S.C. with 19. 7, 7 and 1 year of experience in this field).

In each patient, we evaluated (1) size; (2) shape; (3) internal architecture can be subdivided into (a) necrosis, (b) calcification, (c) Presence of hilum; (4) echogenicity; and (5) nodal border with gray scale US. The short axis and the long axis of the lymph nodes were measured. The shape of the lymph node was determined by the short axis to long axis ratio (S/L). An S/L ratio less than 0.5 indicates an oval node. An S/L ratio greater than or equal to 0.5 indicates a round node.

The vascular distributions of lymph nodes were classified into four categories (1) avascular: absence of flow signals within the LN; (2) peripheral: vascular signals along the periphery of the LN; (3) central: vascular signals branching from central location or radially branching patterns even from the periphery; and (4) mixed: presence of both peripheral and central flows. We evaluated the number of vascular pedicles into four catergories (1) absent; (2) a single vascular signal; (3) a few, less or equal to 5 vascular signals; and (4) multiple, vascular signals more than 5 in a LN.

We categorized total five SWE patterns of SW speed maps according to a method that had been commonly used in prior strain elastography studies on cervical LN^29^ (Fig. 2): (1) absent or very small red (stiff) area; (2) small scattered red areas, total red area less than 45%; (3) large red area, equal or more than 45%; (4) peripheral red are and central green (soft) area, suggesting central necrosis; and (5) red area with or without a green rim. We calculated maximum elasticity (kPA), maximum speed (m/s) and relative ratio (compared to an ipsilateral sternocleidomastoid muscle or an adjacent neck muscles) with several round-shaped ROIs within the stiffest portion of the most of LN.

In differential diagnosis, three head and neck radiologists (I.R., H.J. and S.C.), who were blinded to the final pathology and clinical information except for patients’ age and sex, independently verified sonographic findings of all study groups. They diagnosed each LN with conventional B mode and Doppler US first. After that, they classified SWE patterns of nodes and were asked if they would change their former diagnosis. Cervical LAP with internal necrosis is diagnosed with metastasis and LN with reticulation is regarded as lymphoma. In terms of SWE, softer appearing nodes are diagnosed as lymphoma. Readers asked to make their decision based on their experience when there is a nonspecific LN without typical image findings

Finally, a senior author (S.S.) who had not previously read any of the examinations reviewed all reports and data-collection forms.

### Statistical analysis

Statistical analyses were performed using SPSS 18.0 Statistical Software for Windows (IBM, Armonk, NY, USA). A p value < 0.05 was considered to indicate statistical significance.

Fisher’s exact test and the χ2 test were used to calculate the significance of the difference of categorical variables such as shape, internal architecture, echogenicity, nodal border, vascular distributions, number of vascular pedicles, and SWE pattern between the metastatic LAP and lymphoma groups. Student t-test was used to compare continuous variables such as size of LN, SW elasticity, ratio of SW elasticity and ratio of SW speed. The Mann- Whitney U test was used to compare the SW speed of metastasis and lymphoma.

And then, we calculated Kendall’s Coefficient of Concordance to evaluate the level of agreement for assessing SWE patterns of LN between the three radiologists.

Lastly, we evaluated the diagnostic utility of SWE for differentiating metastatic LAP from lymphoma using receiver operating characteristic (ROC) curve analysis. We presented optimal cut-off values with sensitivity, specificity, positive predictive value (PPV), negative predictive value (NPV) and diagnostic accuracy.
